# Problems recruiting and retaining postnatal women to a pilot randomised controlled trial of a web-delivered weight loss intervention

**DOI:** 10.1186/s13104-018-3305-x

**Published:** 2018-03-27

**Authors:** Anna Haste, Ashley J. Adamson, Elaine McColl, Vera Araujo-Soares, Ruth Bell

**Affiliations:** 1 Institute of Health and Society, Newcastle University, Baddiley Clark Building, Richardson Road, Newcastle upon Tyne, NE2 4AX UK; 2 Human Nutrition Research Centre, Newcastle University, Newcastle upon Tyne, NE2 4HH UK; 3 Fuse, UKCRC Centre for Translational Research in Public Health, Newcastle University, Newcastle upon Tyne, NE2 4AX UK

**Keywords:** Recruitment, Postnatal women, Weight loss, Web, Feasibility, Acceptability

## Abstract

**Objective:**

This paper highlights recruitment and retention problems identified during a pilot randomised controlled trial and process evaluation. The pilot trial aimed to evaluate the feasibility and acceptability of a web-delivered weight loss intervention for postnatal women and associated trial protocol.

**Results:**

General practice database searches revealed low rates of eligible postnatal women per practice. 16 (10%) of the 168 identified women were recruited and randomised, seven to the intervention and nine to the control. 57% (4/7) of the intervention women completed 3 month follow-up measurements in comparison to 56% (5/9) in the control group. By 12 months, retention in the intervention group was 43% (3/7), with 2/7 women active on the website, in comparison to 44% (4/9) of the control group. Interview findings revealed the web as an acceptable method for delivery of the intervention, with the suggestion of an addition of a mobile application. Alternative recruitment strategies, using health visitor appointments, midwifery departments or mother and baby/toddler groups, should be explored. Greater involvement of potential users should enable better recruitment methods to be developed.

*Trial registration* ISRCTN: ISRCTN48086713, Registered 26 October 2012

## Introduction

Postpartum weight retention is of significant concern due to the increased risk of overweight and obesity [1–4]. One year after childbirth 36% of women moved up one or more body mass index (BMI) category [5]. Weight gain in pregnancy does not just affect the pregnancy itself but may contribute to the development of obesity and morbidity in the future [3, 4, 6–8]. Weight loss between pregnancies is encouraged for women with overweight and obesity as this may improve subsequent pregnancy outcomes [9].

Pregnant and postnatal women report interest in accessing diet and physical activity interventions [10], welcoming opportunities to discuss weight change with health professionals [11]. Systematic reviews have identified that combined diet and activity interventions offered in the postnatal period can result in weight loss and reduce postnatal weight retention [12–14]. Further trials are ongoing, one examining a face-to-face feasibility trial involving Slimming World, a commercial United Kingdom (UK) based weight loss organisation that provides lifestyle weight management programme, exploring group weight management after pregnancy [15]. The second trial is a pilot randomised controlled trial using short messaging service delivering a weight management intervention for women who are overweight or obese after pregnancy [16].

Face-to-face interventions for postnatal women are often problematic in terms of childcare, time constraints and returning to work [17, 18]. Individualised programmes which can be accessed flexibly, particularly from home, via telephone or web may be more feasible and successful [14, 19, 20].

Web-delivered weight loss interventions can make access more convenient [21] whilst maintaining privacy and anonymity [22]. A review identified improved weight loss via web-delivered interventions by providing personalised feedback to participants [23]. Web-delivered interventions are under-researched in postnatal populations [13].

The objective of this study was to evaluate the feasibility and acceptability of a web-delivered weight loss intervention, and associated trial protocol, in postnatal women.

## Main text

### Methods

A two arm parallel group rehearsal pilot randomised controlled trial (RCT) with embedded process evaluation was conducted. A detailed account of the intervention and trial protocol is published elsewhere [24].

Women (≥ 18 years of age) who gave birth ≥ 3 months but < 2 years prior who had a BMI ≥ 30 and < 40 kg/m^2^ and were not currently pregnant were eligible to participate in the study. Participants were required to have access to the web and understand English, in order to read the study information sheet, provide informed consent and be able to understand and actively engage with the intervention. The suggested sample size for pilot trials is 30 participants per arm [25, 26], therefore the target sample size in this study was 60.

General practices within County Durham and Darlington (North East England) were invited to take part in the study. Recruited practices conducted patient database searches to identify eligible women. The strategy for recruitment was via invitation letters, signed by the general practitioner (GP), which were sent to potentially eligible participants, who could respond by post, telephone or email. Exercise or dietitian referral occurs within GP practices, therefore we wished responsibility for recruitment to remain within primary care. Letters were sent out via the GP enabling confidentially to be maintained until the patient wished to take part in the study. Baseline appointments checked eligibility and gained informed consent. Baseline measurements were conducted with participants individually randomised with 1:1 allocation using the Sealed Envelope™ system to either the control group or the web-delivered intervention [27].

A key component of the intervention, the My Dietitian website [27], was web-delivered consultations with dietitians and exercise experts, embedded within the website. The website also enabled recording of physical activity levels, food intake by participants, and provided access to recipes, articles and a chat room. The website, developed in Denmark, has previous successful weight loss [28], and was adapted for use in the National Health Service (NHS) in England.

The control arm experienced usual care for weight loss as per the normal practice in their general practice. Taking part in the study did not influence what usual care was offered to the patient.

As this was a pilot trial feasibility and acceptability are suggested as the primary outcome measures [29]. Outcomes measurements included rates of eligibility, invitation responses, declines, consent, randomisation and retention. Quantitative data were analysed using SPSS Statistics version 21.0 software. The parallel process evaluation assessed adherence levels via website usage measures and acceptability of the intervention via semi-structured interviews with participants [30]. The interviews were conducted in English, by the researcher (AH). The topic guide was prepared by reviewing previous research on this topic and ensuring that key details were explored.

Qualitative data (interviews) was recorded, transcribed, imported into QSR NVivo 10 Software and analysed using the five steps of framework analysis [31, 32]: (1) familiarization, (2) identifying a thematic framework, (3) indexing, (4) charting and (5) mapping (interpretation). Two researchers were involved in the analysis stage to validate prominent thematic codes.

### Results

#### Pilot RCT

Eleven GP practice participated. Practice size ranged from 1663 to 19,976 patients, with total registered population of 99,264.

**Fig. 1:**
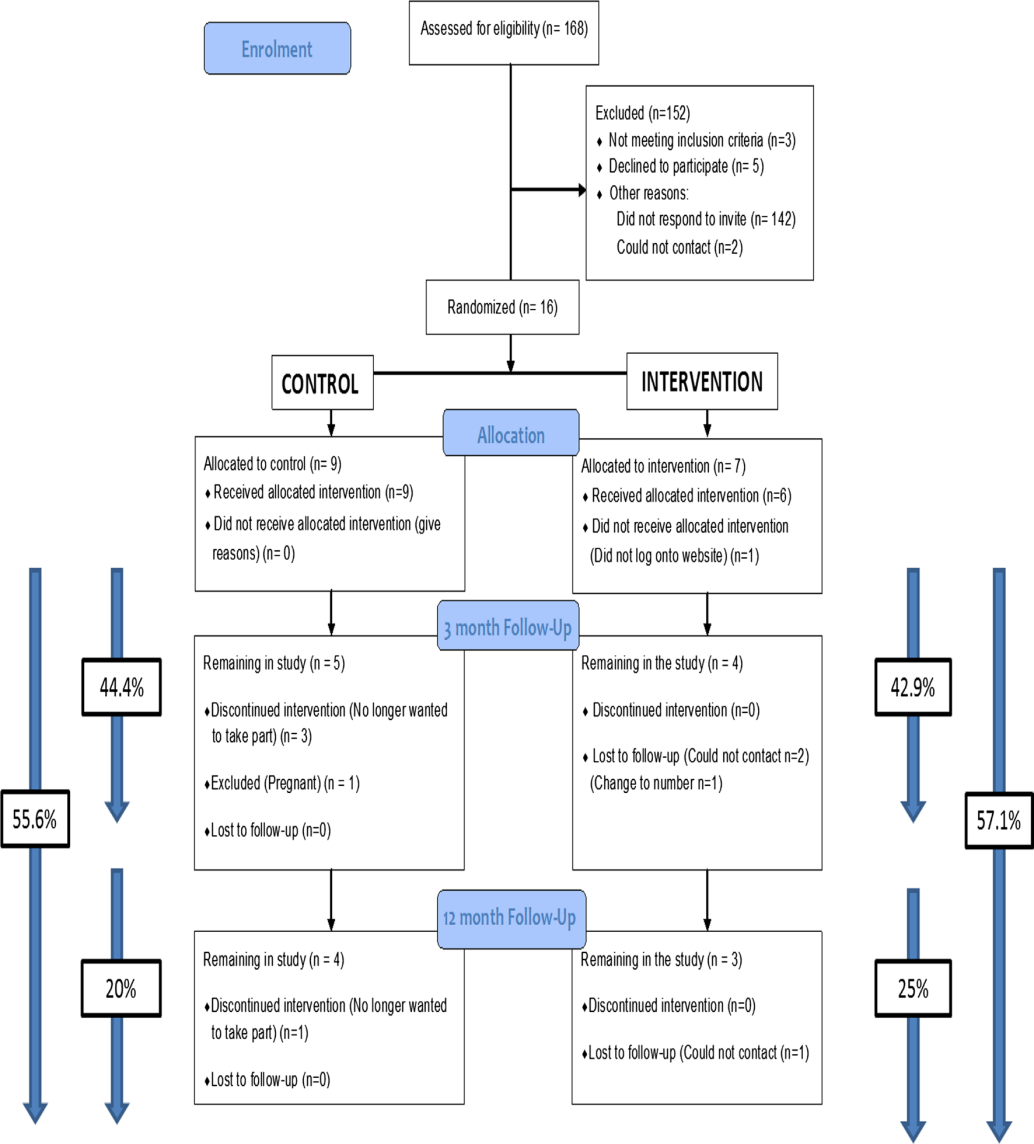
CONSORT pilot RCT participant flow diagram. % included at the sides of the figure show rates of attrition during the study (denominator = number allocated, nominator = number remaining)

168 potentially eligible women were identified and were sent an opt-in recruitment letter. A Consolidated Standards of Reporting Trials (CONSORT) diagram for the study is shown in Fig. 1.

19 women expressed interest in joining the study (11%). Of these, 16 women (10%) were recruited, with three (2%) ineligible due to BMI > 40 kg/m^2^.

5/168 (3%) women declined participation. The remainder did not respond to the letter (142/168 (85%)).

9/16 women (56%) women completed 3 month follow-up measurements. By 12 months, 7/16 (44%) of the women remained. Reasons for non-retention was inability to contact participants (4/16, 25%), participant drop-outs (4/16, 25%) and a participant becoming pregnant (1/16, 6%).

Usual care was the same in all participating practices, with participants in the usual care group receiving no weight loss specific care.

#### Use of website

Four of the seven intervention participants logged on to the ‘My Dietitian’ website, while three (43%) never used the website. Four were still actively using the website at 3 months. At 12 months three remained in the study, but only two were still using the website. The three non-users did not attend 3 month follow up.

Table 1 shows website usage regarding food intake and exercise levels and interactions with allocated dietitians and exercise experts.

**Table 1:** Website usage data averages per intervention participant

Website usage (number of transactions)	3 month website usage		12 month website usage	
	Median (LQ–UQ)	Range (min–max)	Median (LQ–UQ)	Range (min–max)
Dietitian and exercise expert initiated consultations	13 (11–13)	3 (11–14)	22 (19–23)	7 (17–24)
Dietitian initiated consultations	11 (10–12)	3 (10–13)	20 (18–22)	6 (16–22)
Exercise expert initiated consultations	1 (1–2)	1 (1–2)	1 (1–3)	2 (1–3)
Participant initiated messages	17 (2–49)	101 (0–101)	19 (2–59)	141 (0–141)
Food related messages	17 (2–43)	77 (0–77)	19 (2–52)	110 (0–110)
Exercise related messages	0 (0–6)	24 (0–24)	0 (0–8)	31 (0–31)
Food intake entries	2 (1–34)	40 (1–41)	2 (1–37)	40 (1–41)
Exercise entries	1 (0–26)	27 (0–27)	1 (0–28)	35 (0–35)
Log ins	–	–	29 (2–71)	90 (1–91)

The dietitians and exercise experts delivered fewer than intended consultations: a median of 13 out of the proposed 15 at 3 month and 22 out of the proposed 27 at 12 months. The dietitians delivered a greater proportion of scheduled consultations than exercise experts. Participant initiated messages were optional website features. However, participants were encouraged to complete at least one food intake entry and one exercise entry each week (at least 13 by 3 months and 52 by 12 months).

##### Interviews

Five women agreed to be interviewed. A key theme emerging from the interviews was engagement with the intervention and the practicality of it being web-delivered. The women identified how a web-delivered intervention would fit into daily life easily owing to the high convenience of the internet.

*‘nowadays with the modern technology on the phones it’s immediately accessible and erm it’s sort of you don’t have, it’s not hassle if that makes sense it fits in with your lifestyle’ (Participant 15).*

A second theme was the possibility of future developments to improve the intervention. One improvement the women thought could potentially increase efficiency was to incorporate the website into a mobile phone application.

*‘if it was on my phone* – *I’m more on my phone than I am on the real computer’ (Participant 5).*

Lastly, the third theme related to recruitment and potential opportunities within postnatal women. Receiving invitation letters via their GP practices was viewed as appropriate.

*‘I think lends more weight to it because, because it came through the GP, I assumed that it was all gone fine, or absolutely a great thing to do’ (Participant 16).*

Although this recruitment technique was deemed acceptable by these women, it was not successful in terms of recruiting the target number for this study. Therefore ways to try and improve recruitment or identification of eligible women were discussed.

*‘like at the centre you go to [Sure Start] yeah you take, it’s normally the first month or so, I was there quite often’ (Participant 7).*

The women were consistent in their belief that it would be possible and acceptable to inform women of weight loss interventions relatively quickly after childbirth. Suggestions included combining with services that women would already be visiting/receiving, such as recruitment letters in free baby packs, health visitors, midwifery services or mother and baby/toddler groups.

### Discussion

The recruitment target was not met, which was attributable to both low rates of potentially eligible women identified from the GP patient databases and a low uptake among those invited. However, interview findings identified a web-delivered weight loss intervention as feasible and acceptable. A mobile phone application was suggested to potentially improve adherence and retention issues. Comprehensive user engagement is advocated by current intervention development frameworks [33, 34] and could potentially enable better recruitment methods to be developed.

This pilot study achieved a recruitment rate of 10% of 168 invited to participate, with the large majority not responding to the invitation (85%). Other postnatal weight management studies have also reported difficulties with recruitment and retention [35–38], with recruitment ranging from 7 to 28% [39]. Previous recruitment strategies using visits to community groups and posters in community settings have identified higher recruitment rates of 37% [40]. This supports other research which identified the most effective strategy as recruiting via communities [41, 42]. Multi-level approaches such as telephone reminders, incorporating into clinical flow and the use of opt-out recruitment have been suggested to increase recruitment [43, 44].

An ongoing feasibility trial has incorporated opt out recruitment using maternity administration systems to identify eligible patients [15]. Opt out methods have been shown to produce higher response and recruitment than opt-in techniques [44, 45] but at present are not favoured by ethics committees, owing to controversy over contacting participants before receiving their permission for their personal details to be accessed.

Attrition rates have been shown to vary from 0 to 42% [35, 36, 40, 46]. Lower attrition rates have been linked to integration into existing services, the provision of incentives or having access to the intervention at home [2, 17, 42, 43], linking to findings identified within this study’s interviews.

### Conclusion

Web-delivered weight loss interventions have potential in this group with the process evaluation identifying the intervention as feasible and acceptable. A definitive trial would require significant modification to the study design, primarily in relation to the strategies used to identify and recruit postnatal women. Further work is needed to determine successful recruitment strategies.

Midwifery departments, health visitors or mother and baby/toddler groups may be possible pathways to reach a larger number of postnatal women as supported by prior literature [40, 41] and the interview findings within this study. Research is lacking on interventions to reduce postnatal weight retention and further research is needed to identify the best approaches to recruitment and retention.

### Limitations

A limitation of this study was the below target sample size achieved. Potential reasons for this may have been due to the GPs identifying lower numbers (168) of postnatal women within the included practices than would be typically expected (265–352) based on general fertility rates. A possible explanation may be practices having fewer women of child-bearing age (17%) than the UK general practice population (20%) [47] and therefore fewer pregnancies. A possible solution would be to target practices most likely to have larger numbers of obese postnatal women. Research active practices were contacted to take part in this study, unfortunately it is not known how many practices were contacted by the Clinical Research Network.

Incomplete practice recording of BMI in this population meant three women (16% of those assessed at baseline) had BMI > 40, too high for inclusion, even though GP database records showed their BMI to be eligible.

Another study limitation was the number of participants who dropped out during the study timeframe. It was not possible to interview women who had not engaged or dropped out to identify reasons why. Ethical approval restricted the ability to interview study decliners.

## Abbreviations

BMI: body mass index
CONSORT: Consolidated Standards of Reporting Trials
GP: general practitioner
NHS: National Health Service
RCT: randomised controlled trial
UK: United Kingdom
